# Tocotrienol-Rich Fraction, [6]-Gingerol and Epigallocatechin Gallate Inhibit Proliferation and Induce Apoptosis of Glioma Cancer Cells

**DOI:** 10.3390/molecules190914528

**Published:** 2014-09-12

**Authors:** Amirah Abdul Rahman, Suzana Makpol, Rahman Jamal, Roslan Harun, Norfilza Mokhtar, Wan Zurinah Wan Ngah

**Affiliations:** 1 UKM Medical Molecular Biology Institute (UMBI), UKM Medical Center, Jalan Ya’acob Latiff, Bandar Tun Razak, Cheras 56000, Malaysia; 2 Department of Biochemistry, Faculty of Medicine, Universiti Kebangsaan Malaysia, Jalan Ya’acob Latiff, Bandar Tun Razak, Cheras 56000, Malaysia

**Keywords:** tocotrienol-rich fraction, [6]-gingerol, epigallocatechin gallate, asiaticoside, synergy, glioma

## Abstract

Plant bioactives [6]-gingerol (GING), epigallocatechin gallate (EGCG) and asiaticoside (AS) and vitamin E, such as tocotrienol-rich fraction (TRF), have been reported to possess anticancer activity. In this study, we investigated the apoptotic properties of these bioactive compounds alone or in combination on glioma cancer cells. TRF, GING, EGCG and AS were tested for cytotoxicity on glioma cell lines 1321N1 (Grade II), SW1783 (Grade III) and LN18 (Grade IV) in culture by the (3-(4,5-dimethylthiazol-2-yl)-5-(3-carboxymethoxy-phenyl)-2-(4-sulfophenyl)-2H-tetrazolium, inner salt) (MTS) assay. With the exception of AS, combinations of two compounds were tested, and the interactions of each combination were evaluated by the combination index (CI) using an isobologram. Different grades of glioma cancer cells showed different cytotoxic responses to the compounds, where in 1321N1 and LN18 cells, the combination of EGCG + GING exhibited a synergistic effect with CI = 0.77 and CI = 0.55, respectively. In contrast, all combinations tested (TRF + GING, TRF + EGCG and EGCG + GING) were found to be antagonistic on SW1783 with CI values of 1.29, 1.39 and 1.39, respectively. Combined EGCG + GING induced apoptosis in both 1321N1 and LN18 cells, as evidenced by Annexin-V FITC/PI staining and increased active caspase-3. Our current data suggests that the combination of EGCG + GING synergistically induced apoptosis and inhibits the proliferation 1321N1 and LN18 cells, but not SW1783 cells, which may be due to their different genetic profiles.

## 1. Introduction

Despite recent advances in medicine, mortality from malignant astrocytic gliomas, representing the most common primary tumors of the brain, still remains unacceptably high, partly due to chemo-resistance and recurrence [1]. In addition, aggressive chemotherapy is usually associated with debilitating toxic side effects. Therefore, the search for alternative preventive and therapeutic strategies continues to be an important goal.

Non-cytotoxic natural products possess pleiotropic properties and represent a possible therapeutic approach for cancers, including brain cancer. To date, most mechanistic studies on dietary chemopreventive agents have utilized single dietary agents at high concentrations, which are unlikely to be achieved by food intake [2]. The use of bioactives in combination represents an alternative approach and can be explored with the potential to be used as an adjuvant therapy or in the prevention of recurrence. Before further investigation of combination therapy can be tested in clinical trials, which are known to be expensive and time-consuming, biomedical studies, such as *in vitro* screening and quantification of synergy, need to be done to generate fast and robust data [3].

Bioactive compounds with similar effects will sometimes result in exaggerated or diminished effects when used simultaneously. Synergistic interaction can be achieved if the constituents of compound mixtures affect distinct targets or interact with one another to improve the solubility and, in turn, enhance the bioavailability of one or several substances of the multi-compound combination. Hypothetically, a combination of compounds can affect several targets, such as enzymes, substrates, metabolites and proteins, receptors, ion channels, DNA/RNA, monoclonal antibodies, signal cascades and physicochemical mechanisms [4]. Thus, the use of compounds in combination may target complementary sites of action, resulting in the inhibition of the proliferation of cancer cells.

It is well established that cancer can be prevented by healthy eating habits, particularly of fruits and vegetables; possibly as a result of the synergistic interaction between low-dose phytochemicals and micronutrients, for which little information or evidence currently exists [5]. In this study, the concept of synergistic interaction was tested by examining individual compounds and combinations of two plant bioactives, [6]-gingerol (GING), epigallocatechin gallate (EGCG) and asiaticoside (AS), which are frequently found in a traditional Asian diet, and a vitamin E isomer mixture, tocotrienol-rich fraction (TRF), against glioma cancer cell lines. Each chosen compound has been reported to show anti-cancer activities, with overlapping and different molecular actions and targets. For example, TRF exerts its antitumor effects by enhancing immune response [6], whereas [6]-gingerol induces apoptosis by affecting the mitochondrial signaling pathway and modulating p53 [7]. EGCG exerts epigenetic control by inhibiting DNA methyltransferases (DNMT) and histone acetyltransferase (HAT) to obstruct tumor cell proliferation [8], an effect not reported for TRF or GING; whereas AS significantly inhibits azoxymethane (AOM)-induced tumorigenesis in the intestines of F344 rats and HepG2 human hepatoma cells, although the inhibitory mechanisms of AS are not fully understood [9].

Since each of these natural compounds possesses their own specific activities, the aim of this study is to investigate the interactions of the compounds by exposing different grades of glioma cells to a sub-effective dose of each compound combined, followed by the determination of cell proliferation and apoptosis by the presence of caspase-3 and Annexin-V FITC/PI. We report the effect of TRF, GING and EGCG alone and in combinations of two on Grades II, III and IV glioma cells. The different interaction indices obtained from an isobologram will provide information on the type and size of interactions between the combinations on the different cell lines.

## 2. Results and Discussion

### 2.1. Effect of Bioactives on the Viability of Glioma Cells

**Figure 1:**
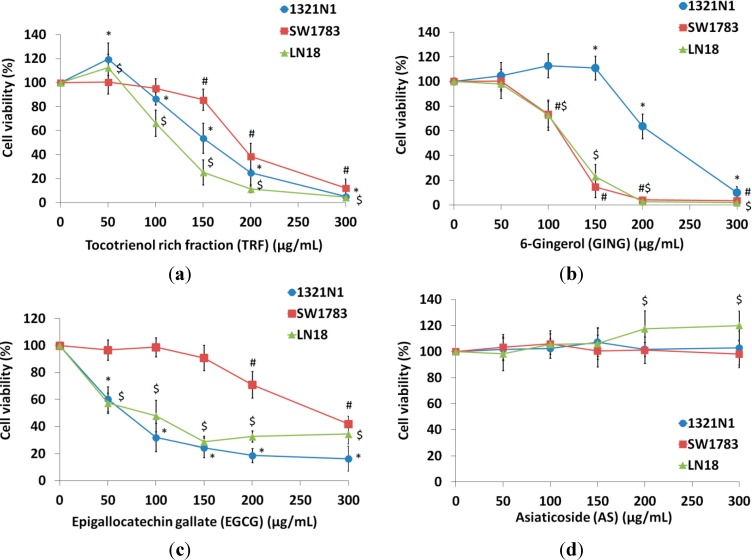
Treatment of (**a**) tocotrienol-rich fraction (TRF), (**b**) [6]-gingerol (GING), (**c**) epigallocatechin gallate (EGCG) and (**d**) asiaticoside (AS) on 1321N1, SW1783 and LN18 for 24 h. The cell survival test was determined by the MTS assay. Data are presented as the means ± SD, *n* = 9.

All of the compounds tested, with the exception of AS, inhibited the growth of 1321N1, SW1783 and LN18 cells with inhibitory concentration at 50% cell death (IC_50_) values ranging from 142–202 µg/mL for TRF, IC_50_ values for GING ranging from 132–243 µg/mL, while the IC_50_ values for EGCG were from 82–302 µg/mL (Table 1). Cytotoxicity induced by TRF and GING was found to be dose dependent with almost 90% inhibition achieved after 24 h of treatment. However, the percentages of growth inhibition by EGCG against all cell lines at the highest concentration (300 µg/mL) were only from 50%–80% (Figure 1). Interestingly, no significant changes in cell proliferation were observed on all cell lines treated with AS. Therefore, AS was not tested further.

**Table 1:** (3-(4,5-Dimethylthiazol-2-yl)-5-(3-carboxymethoxy-phenyl)-2-(4-sulfophenyl)-2H-tetrazolium, inner salt) (MTS) cytotoxic effect of natural bioactives on human glioma cancer cells (1321N1, SW1783, LN18). Viable cells (%) were expressed as the mean ± SD of three independent experiments.

Cell Lines	Compound	IC_50_ Value (µg/mL)	Viability (% Cells) ^a^
Grade II 1321N1	Tocotrienol rich fraction 70 (TRF)	171.5 ± 11.43	5.1 ± 1.1
	Epigallocatechin gallate (EGCG)	82.0 ± 10.31	16.3 ± 9.2
	[6]-gingerol (GING)	243.0 ± 11.6	10.0 ± 5.0
	Asiaticoside (AS)	n/a	102.8 ± 11.1
Grade III SW1783	Tocotrienol rich fraction 70 (TRF)	202.0 ± 6.02	12.3 ± 7.5
	Epigallocatechin gallate (EGCG)	300.0 ± 9.10	41.9 ± 5.7
	[6]-gingerol (GING)	132 ± 4.51	3.5 ± 2.0
	Asiaticoside (AS)	n/a	98.1 ± 10.3
Grade IV LN18	Tocotrienol rich fraction 70 (TRF)	142.0 ± 5.03	4.5 ± 1.9
	Epigallocatechin gallate (EGCG)	134.0 ± 11.36	34.4 ± 7.4
	[6]-gingerol (GING)	132.5 ± 10.11	1.8 ± 0.9
	Asiaticoside (AS)	n/a	120.1 ± 11.2

^a^ The percentage of cell viability after 24-h incubation of the maximum concentration treatment (300 µg/mL) of each compound.

* *p* < 0.05 compared to the untreated 1321N1; # *p* < 0.05 compared to the untreated SW1783; $ *p* < 0.05 compared to the untreated LN18.

### 2.2. Isobologram Analysis of the Interaction between Bioactive Compounds

**Figure 2:**
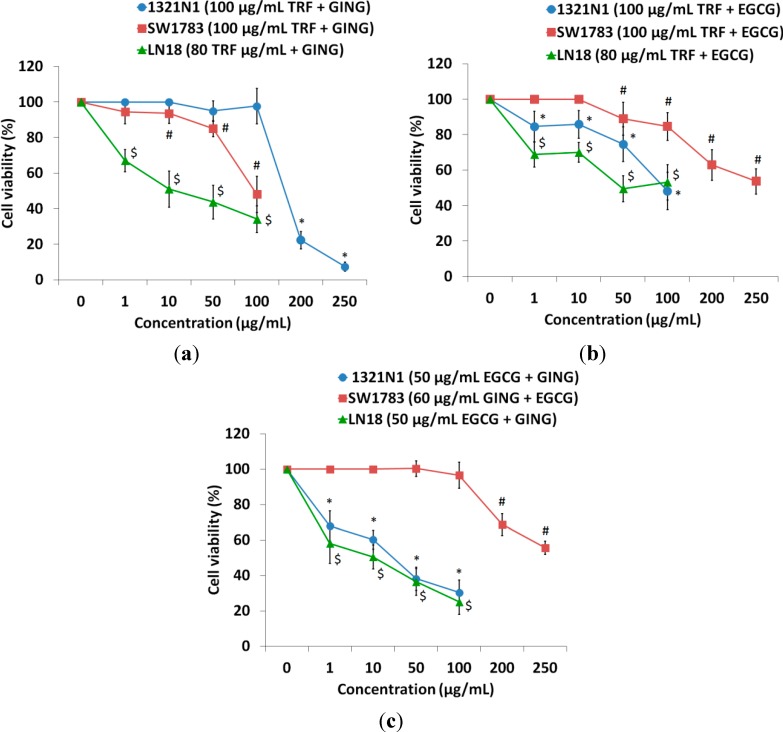
Combined treatment of (**a**) TRF with GING, (**b**) TRF with EGCG and (**c**) EGCG with GING on 1321N1, SW1783 and LN18 for 24 h. The cell survival test was determined by the MTS assay. Data are presented as the means ± SD, *n* = 9.

The goal of this investigation was to determine whether combining compounds TRF, GING and EGCG would induce synergistic interaction in inhibiting the proliferation of glioma cell lines at lower doses. Hence, we examined the effects of increasing doses of each agent alone or in combination on the growth of glioma cell lines (Figure 1 and Figure 2). The growth inhibition of cells affected by combinations of bioactives was greater than treatment with individual bioactives. Interactions between compounds were analyzed by obtaining the combination index (CI) using an isobologram plot. Values of CI < 0.7 are indicative of strong synergism or synergism, while 0.7 < CI < 0.9 suggests moderate or slight synergism; and CI > 0.9 showed a nearly additive effect or antagonism. Synergism was observed in the combined treatment of a sub-effective dose of EGCG with GING in 1321N1 and LN18 cells (Table 2); while a moderate synergistic interaction was shown when TRF was used in combination with GING on LN18 cells. A combination of TRF with EGCG was found to be antagonistic on all three glioma cancer cells (Table 2). All subsequent experiments were carried out on combined treatments showing a synergism of CI < 1.0 only.

* *p* < 0.05 compared to the untreated 1321N1; # *p* < 0.05 compared to the untreated SW1783; $ *p* < 0.05 compared to the untreated LN18.

### 2.3. Effect of Combined Bioactives on Apoptosis

**Figure 3:**
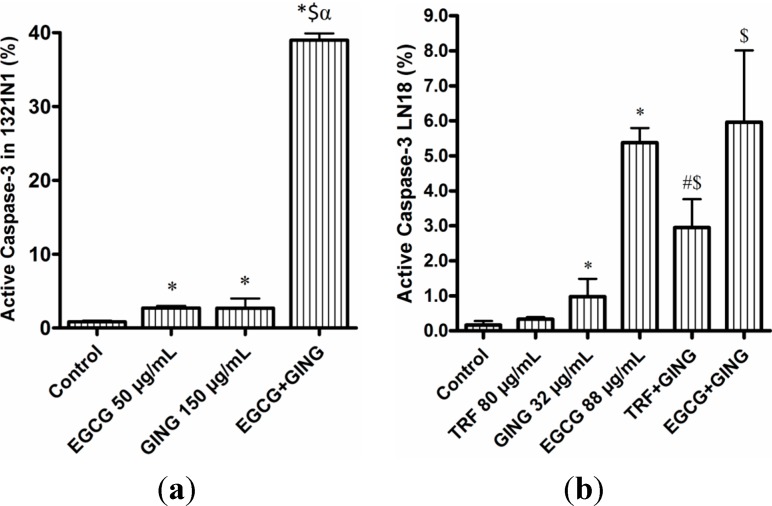
Combined compounds caused greater inhibition of growth of (**a**) 1321N1 and (**b**) LN18 cells than either agent alone, as evidenced by the presence of active caspase-3. Each value represents the mean ± SD of three independent experiments.

The effect of combined compounds with synergistic interaction in the apoptosis of glioma cancer cells 1321N1 and LN18 was further studied using FITC Active Caspase-3 Apoptosis Kit and FITC Annexin-V Apoptosis Detection Kit to identify the presence of apoptotic cells after 24 h of treatment. The synergistic effect of combined EGCG + GING on 1321N1 resulted in increased caspase-3 activation (39.2%) compared to treatment by either EGCG or GING alone (Figure 3a). However, no significant differences in the induction of active caspase-3 were observed in LN18 treated with EGCG alone (5.4%) or EGCG + GING (6%) in combination (Figure 3b). Whereas for combined treatment of TRF with GING on LN18, a slight increase of active caspase-3 (3%) compared to individual compounds was observed (Figure 3b).

**Table 2:** The ratio of combined compounds at a growth inhibition of 50% (IC_50_) on glioma cancer 1321N1, SW1783 and LN18 cells and the combination index (CI) for each combination. The data are the average of three independent experiments.

**Type of Cell Line**	**TRF:GING**	**IC_50_^a^ (µg/mL)**	**TRF ^b^ (µg/mL)**	**GING ^b^ (µg/mL) **	**Combination Index ^c^ (CI)**
1321N1	5:8	160 ± 4.36	171.5 ± 11.43	243 ± 11.60	1.24
SW1783	10:11	110 ± 8.89	202 ± 6.02	132 ± 4.51	1.29
LN18	5:2	32 ± 10.21	142 ± 5.03	132.5 ± 10.11	0.80
**Type of Cell Line**	**TRF:EGCG**	**IC_50_^a^ (µg/mL)**	**TRF ^b^ (µg/mL)**	**EGCG ^b^ (µg/mL)**	**Combination Index ^c^ (CI)**
1321N1	1:1	100 ± 9.5	171.5 ± 11.43	82 ± 10.31	1.80
SW1783	10:27	270 ± 4.16	202 ± 6.02	300 ± 9.10	1.39
LN18	10:11	88 ± 11.14	142 ± 5.03	134 ± 11.36	1.22
**Type of Cell Line**	**EGCG:GING**	**IC_50_^a^ (µg/mL)**	**EGCG ^b^ (µg/mL)**	**GING ^b^ (µg/mL)**	**Combination Index ^c^ (CI)**
1321N1	5:4	40 ± 8.62	82 ± 10.31	243 ± 11.60	0.77
SW1783	9:2	60 ± 5.6	300 ± 9.10	132 ± 4.51	1.35
LN18	2:1	24 ± 2.65	134 ± 11.36	132.5 ± 10.11	0.55

^a^ IC_50_ of the combined compounds; ^b^ IC_50_ of compounds A or B; ^c^ CI < 1.0 indicates synergism; 0.9 < CI < 1.10 indicates a nearly additive effect; CI > 1.10 indicates antagonism.

* *p* < 0.05 compared to the control; # *p* < 0.05 compared to TRF; $ *p* < 0.05 compared to GING; α *p* < 0.05 compared to EGCG.

**Figure 4:**
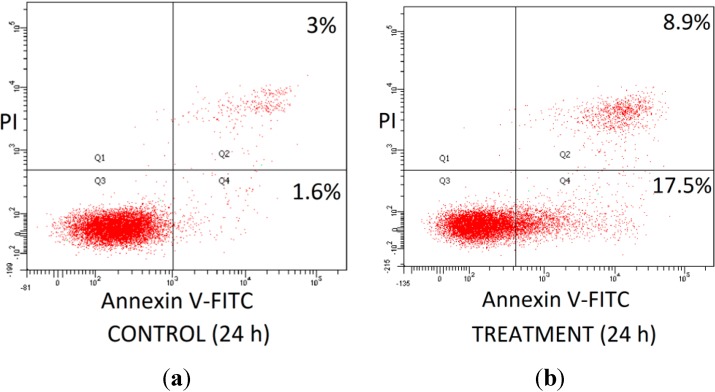
Detection of apoptosis using flow cytometry after annexin V-FITC/propidium iodide (PI) staining for (**a**) Control and (**b**) Treatment. Viable cells are in the lower left quadrant (Q3); early apoptotic cells are in the lower right quadrant (Q4); late apoptotic cells are in the upper right quadrant; and non-viable necrotic cells are in the upper left quadrant (Q1).

**Figure 5:**
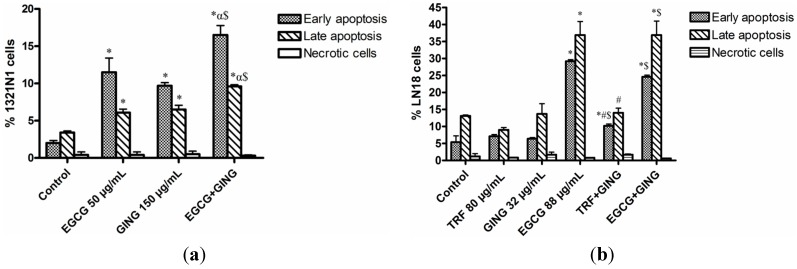
Combined treatments potentiate apoptosis-mediated cell death in 1321N1 and LN18 cells.1321N1 and LN18 cells were treated with combined treatments at IC_50_ concentrations for 24 h. Bar graph representing mean values from three independent experiments for (**a**) 1321N1 and (**b**) LN18.

Similar results were obtained for double fluorescence staining of the Annexin-V FITC/PI flow cytometry assay (Figure 4). As shown in Figure 5a, the percentage of both early and late apoptotic cells for the EGCG + GING treatment in 1321N1 cells increased significantly (*p* < 0.05) compared to EGCG or GING treatment alone. Early and late apoptosis were increased in LN18 cells treated with combined TRF + GING and EGCG + GING when compared to TRF or GING alone, respectively (Figure 5b). A similar observation as active caspase-3 (Figure 3b) was obtained on early and late apoptotic LN18 cells treated with EGCG + GING when compared with EGCG alone (Figure 5b).

* *p* < 0.05 compared to the control early or late apoptosis, respectively; # *p* < 0.05 compared to the TRF early or late apoptosis, respectively; $ *p* < 0.05 compared to the GING early or late apoptosis, respectively; α *p* < 0.05 compared to the EGCG early or late apoptosis, respectively.

Accumulating evidence suggests that EGCG, TRF, GING and AS have the potential to impact a variety of human diseases, such as cancer [10,11,12] and neurodegeneration [13]. The individual cytotoxic effects of EGCG, TRF and GING on several cancer cells *in vitro*, such as the effects of EGCG on colon cancer cells [14], GING on mutant p53-expressing pancreatic cancer cells [15] and the effect of tocotrienols on breast cancer cells [16], have been reported. However, not much is known about the effects of these bioactives in combination on glioma cancer cells.

TRF, GING and EGCG consistently inhibit cell proliferation in Grade II 1321N1, Grade III SW1783 and Grade IV LN18 glioma cells, although higher concentrations of GING were needed for 1321N1 cells (Figure 1b) compared to other grades of glioma cells in this study, while a much higher dose of EGCG was required to inhibit the proliferation of SW1783 cells compared to 1321N1 and LN18 cells (Figure 1c). Different characteristics of 1321N1, SW1783 and LN18 cell lines were observed in previous studies. For example, 1321N1 cells contain M2-gland muscarinic receptors [17], while some of the characteristics of SW1783 include harboring an amplified *PDGFRA* (4q11) gene, possessing wild-type *CDKN2A*, the loss of one copy of *PIK3CA* (3q26.3), the low-level copy number gain of *BIRC5* (17q25) [18] and showing a lack of the *PTEN−* gene [19]. LN18 was identified to carry a *PTEN+* wild-type gene, *CDKN2A−* [19], *p16−* and *p14ARF−* deleted gene, a *p53+* mutated gene and has been reported to express the *MGMT* gene [18,20,21]. It is most likely that the mechanism of GING and EGCG in inhibiting the proliferation of glioma cells and the differences in the treatment dosage are affected by the presence of different mutations or other specific characteristics involved in different grades of cell lines [22]. Evidence has shown that the mutation status of PTEN phosphatase influenced the response of glioma cell lines to EGFR inhibitors [19]. Furthermore, the apoptotic effects of tocotrienols were shown via the activation of various intracellular signaling mechanisms depending on the type of cancer cells [23].

Interestingly, in this study, the treatment with AS did not affect cell proliferation on 1321N1, SW1783 and LN18 glioma cell lines (Figure 1d, Table 1), which contradicts a recent study on the MCF-7 breast cancer cell line by Al-Saeedi *et al.* [12]. The pattern of AS induction of cell death reported at 24 h and 48 h of AS treatment in Al-Saeedi’s [12] study was similar, with an increase of inhibition by only about 10% after 48 hours of AS treatment. The difference observed could be due to the different type of cancer cells tested and that the characteristics of breast cancer cells confer susceptibility to AS compared to glioma cells. An increase of apoptosis was also shown in AOM-induced tumorigenesis in rats after a treatment of 10 mg/kg of *Centella asiatica* extract, which contains AS. However, *C. asiatica* extract was found to be less effective, mainly in the large intestine at a higher dose of 100 mg/kg [9]. Bunpo *et al.* [9] suggested that AS or other components present in *C. asiatica* perhaps possess the ability to enhance carcinogenesis by inducing angiogenesis or other mechanisms.

Mixtures of several bioactive constituents, such as TRF, can also exhibit synergistic effects by its anti-angiogenic properties, which were found to significantly reduce 4T1 breast tumor volume in BALB/c mice [24]. However, treatment of combined TRF with EGCG results in an antagonistic interaction on all glioma cells tested in this study. Combinations of these compounds can strongly enhance overall efficacy if they are bioavailable, because isomers of vitamin E, especially tocotrienols, possess high affinity for cell membranes and may co-localize and interact with PUFA and cholesterol molecules in the membrane to influence the structure and signaling function of membrane domains [10]. Furthermore, tocotrienols were reported to be bioavailable to all vital organs when taken orally, and different isomers of tocotrienols, such as γ- and δ-tocotrienols, have been reported to inhibit the proliferation of MDA-MB-435 human breast cancer cells [25]. On the other hand, EGCG, which falls into the flavonoid group, possess polyvalence effects that aid in the binding ability to different molecular structures, like enzymes and proteins [26]. Perhaps the antagonistic effect in this mixture of compounds was the result of the binding ability to several different targets or genes in a pathway that cancelled out the effect of each other.

Characteristics of cells undergoing apoptosis can be studied by multiple hallmarks, such as the presence of active caspase-3 and the staining of exposed phosphatidylserine on the cell surface, which is demonstrated during the Annexin-V FITC/PI flow cytometry assay. Observations on the cell proliferation assay and the percentage of early and late apoptotic cells in this study suggest that treatment of 1321N1 and LN18 with combined EGCG + GING and/or TRF + GING induced both anti-proliferative and apoptotic effects. However, the effects of EGCG + GING in terms of caspase-3 activation on LN18 cells in this study was not consistent with past reports on GING [11] or EGCG [27], where it has been shown to regulate the apoptotic signaling pathway via caspase-3 activation in most cancer cells.

Further studies are needed to elucidate additional molecular signaling pathways on other cell death regulation genes, which may be involved following EGCG + GING and TRF + GING treatment on LN18 cells, where synergism interactions occur between the compounds, but are not fully explained by caspase-3 activation. Perhaps, due to the mutation of p53 and the deletion of p16 and p14ARF in LN18, different genes were activated, aside from caspase-3, which contributed to the inhibition of cell proliferation and the induction of apoptosis, as evidenced by Annexin-V FITC/PI staining (Figure 5b). This was observed in a study by Zhang *et al.* [28], where the variation in the induction of apoptosis by combined all*-trans* retinoic acid (ATRA) and/or interferon gamma (IFN-γ) might be attributed to the difference in PTEN expression in LN18 (PTEN-proficient) and U87MG (PTEN-deficient) cells. Another possible explanation would be that the efficacy of treatment was contributed mainly by the inhibition of LN18 cell growth rather than through induction of apoptosis. This was shown in a study by Gupta [29], where their targeted treatment using PPARγ selective compound GW7647 only slowed the proliferation, but did not induce apoptosis of colon cancer cells.

The effectiveness of well-chosen combinations, such as combined EGCG + GING against 1321N1 cells in this study and in activating caspase-3 rather than with individual EGCG or GING compound (Figure 3a), has been proven by a study on prostate cancer [30], where the combinations of EGCG, genistein and quercetin trigger apoptosis in CWR22R*v*1 via multiple actions, not through direct inhibition of the tumor, but by suppression or activation of different processes, which are critical for the tumor’s survival [2].

Tocotrienols [31] and EGCG [32] have been reported to be capable of crossing the blood-brain barrier, while GING were found to be distributed in the brain tissues of rats after oral administration [33]. However, not many studies were found on the bioavailability of GING in the brain. Nonetheless, researchers are now experimenting on several delivery systems, such as nanoparticles, cyclodextrins, niosomes and liposomes, which could deliver a chemopreventive agent to a specific target tissue [34]. In fact, a study by Smith A. *et al.* [35] using nanolipidic EGCG particles reported an increase of the oral bioavailability *in vivo* by more than two-fold.

Synergism of drugs in combination is recognized, and while there was much evidence reporting the anticancer effects of TRF, GING and EGCG, the exact mechanisms involved are less known. Further investigation on the mechanism of action by these combined compounds is needed.

## 3. Experimental Section

### 3.1. Reagents and Chemicals

Tocotrienol-rich fraction (TRF) was purchased from Sime Darby Bioganic Sdn. Bhd. (Selangor, Malaysia), asiaticoside (AS) from Chengdu Biopurify Phytochemicals Ltd (Sichuan, China) and [6]-gingerol (GING) and epigallocatechin gallate (EGCG) from Sigma-Aldrich Co. (St. Louis, MO, USA). The FITC Active Caspase-3 Apoptosis Kit and FITC Annexin V Apoptosis Detection Kit were purchased from BD Biosciences (San Jose, CA, USA). The other chemicals used were all of analytical grade.

### 3.2. Cell Line and Culture Condition

Human glioblastoma cell lines 1321N1 were purchased from the European Collection of Cell Culture (ECACC), while SW1783 and LN18 were obtained from the American Type Culture Collection (Manassas, VA, USA). 1321N1 and LN18 were cultured in Dulbecco’s modified Eagle medium (DMEM) supplemented with penicillin, streptomycin, 10% fetal bovine serum (FBS) and 5% FBS, respectively, in a humidified incubator at 37 °C in an atmosphere of 95% air and 5% CO_2_. SW1783 was maintained in Leibovitz, 10% FBS, in an atmosphere of 100% air. The medium was changed three times a week, and cells were passaged using accutase.

### 3.3. Treatments with Natural Compounds

Stock solutions of TRF and AS were prepared in absolute ethanol, while GING was dissolved in dimethyl sulfoxide (DMSO) and stored at −20 °C. EGCG was prepared fresh in culture medium. As the vehicle, 0.1% of ethanol or 0.5% DMSO was added to control cells.

### 3.4. Determination of Cell Viability

Viability of glioblastoma cancer cell lines treated with the four compounds and their combinations was determined using the CellTiter 96^®^ Aqueous Non-Radioactive Cell Proliferation Assay (Promega, San Luis Obispo, CA, USA) as previously described. Briefly, 1.0 × 10^4^ cells per well were seeded in 96-well microtiter plates (Nunc). After 24 h incubation, the medium was removed, and the cells were treated with 100 μL of medium at final concentrations of 50, 100, 150, 200 and 300 μg/mL of individual compound for 24 h in triplicates and repeated three times. After 24 h incubation, the medium was carefully removed, replaced with fresh medium, and 20 μL of (3-(4,5-dimethylthiazol-2-yl)-5-(3-carboxymethoxy-phenyl)-2-(4-sulfophenyl)-2H-tetrazolium, inner salt) (MTS) was added to each well and incubated at 37 °C for 2 h. Absorbance was measured at 490 nm in a VersaMax ELISA micro plate reader (Molecular Device, Sunnyvale, CA, USA). The percentage of viable cells at each concentration was calculated by dividing the absorbance (A490) of treated cells by that of control cells. The half maximal inhibitory concentration (IC_50_) was determined from the cell viability (%) *vs.* concentrations graph. For compounds in combination, half or a quarter of the IC_50_ of TRF was initially titrated to a range of concentrations of GING or EGCG (1, 10, 50, 100, 250 μg/mL); while half or a quarter of IC_50_ of EGCG was initially titrated to a range of concentrations of GING (1, 10, 50, 100, 250 μg/mL). All assays were performed in triplicates and repeated in three independent experiments.

### 3.5. Active Caspase-3 Apoptosis Assay

The presence of active caspase-3 was determined using the FITC Active Caspase-3 Apoptosis Kit. Cells were plated in 60-mm culture dish at a seeding density of 5 × 10^5^ cells/dish. EGCG, GING or TRF were dissolved in medium, DMSO or ethanol, respectively, and added to the culture media to the final concentration specified. Vehicle alone was also added and served as the untreated control. After 24 h, cells were harvested and washed twice with PBS. Assays were performed as described in the manufacturer’s protocol. Briefly, cells were fixed in BD Cytofix/Cytoperm solution, incubated on ice for 20 min, washed with BD Perm/Wash buffer, and FITC rabbit anti-active caspase-3 antibody was added and incubated for 30 min at room temperature. Fluorescence from a population of 1 × 10^5^ cells was detected using the BD FACSCantoTM flow cytometer (Becton Dickenson, Mountain View, CA, USA) and CellQuest Pro (IVD) software (Becton Dickenson, Mountain View, CA, USA). Experiments were performed in duplicate and repeated three times.

### 3.6. Annexin V-Propidium Iodide Staining Apoptosis Assay

Apoptosis was determined by assessing the membrane changes (phosphatidylserine based) using the FITC Annexin V Apoptosis Detection Kit. Cells were plated in 60-mm culture dish at a seeding density of 5 × 10^5^ cells/dish. Cells in culture were treated with EGCG, GING or TRF, as above. The subsequent procedures for Annexin V/FITC labeling were carried out according to the instructions provided by the manufacturer. Briefly, after 24 h, cells were harvested, washed twice with PBS and resuspended in 1× binding buffer. Annexin-V FITC and propidium iodide (PI) were added and incubated for 15 min at room temperature (25 °C) in the dark. Fluorescence from a population of 1 × 10^5^ cells was detected using the BD FACSCanto^TM^ flow cytometer (Becton Dickenson, Mountain View, CA, USA) and CellQuest Pro (IVD) software (Becton Dickenson, Mountain View, CA, USA). The experiments were performed in duplicate and repeated three times.

### 3.7. Statistical Analysis

The levels of interaction between two bioactives were determined by isobologram analysis based on the Chou–Talalay method [36,37], where the output is represented as combination indexes (CI). The CI between two compounds A and B is:

CI = (C_A,X_/IC_X,A_) + (C_B,X_/IC_X,B_) (1)

Based on CI values, the extent of synergism/antagonism may be determined. In brief, CI values between 0.9 and 0.85 would suggest a moderate synergy, whereas those in the range of 0.7 to 0.3 are indicative of clear synergistic interactions between the compounds. CI values in the range of 0.9 to 1.10 suggest a nearly additive effect. CI values >1.10 suggest antagonistic interactions.

Differences among the various treatment groups in cell viability and apoptosis studies were performed by SPSS 16.0 software using a two-tailed Student’s *t*-test, and *p* < 0.05 was considered statistically significant. The data were expressed as the mean ± standard deviation (SD).

## 4. Conclusions

The combination of EGCG + GING synergistically induced apoptosis in 1321N1 and LN18, representing Grades II and IV glioma cancer cells, but not Grade III SW1783 cells. Enhanced inhibitory effects may be obtained at correctly chosen combinations of natural bioactives, thereby requiring lower concentrations. Moreover, the differing responses to EGCG + GING treatment perhaps depend on the genetic profiles. Further studies are required to elucidate the mechanisms of action mediated by these combined compounds.
